# Discovery of Anticancer Activity of Amentoflavone on Esophageal Squamous Cell Carcinoma: Bioinformatics, Structure-Based Virtual Screening, and Biological Evaluation

**DOI:** 10.4014/jmb.2203.03050

**Published:** 2022-05-04

**Authors:** Lei Chen, Bo Fang, Liman Qiao, Yihui Zheng

**Affiliations:** 1 The Second Affiliated Hospital and Yuying Children's Hospital of Wenzhou Medical University, Wenzhou, Zhejiang 325027, P.R. China; 2 College of Pharmacy, Wenzhou Medical University, Wenzhou, Zhejiang 325015, P.R. China

**Keywords:** Esophageal carcinoma, cyclin B1, cyclin A, amentoflavone

## Abstract

Esophageal squamous cell carcinoma (ESCC) is the most common primary esophageal malignancy with poor prognosis. Here, due to the necessity for exploring potential therapies against ESCC, we obtained the gene expression data on ESCC from the TCGA and GEO databases. Venn diagram analysis was applied to identify common targets. The protein-protein interaction network was constructed by Cytoscape software, and the hub targets were extracted from the network via cytoHubba. The potential hub nodes as drug targets were found by pharmacophore-based virtual screening and molecular modeling, and the antitumor activity was evaluated through in vitro studies. A total of 364 differentially expressed genes (DEGs) in ESCC were identified. Pathway enrichment analyses suggested that most DEGs were mainly involved in the cell cycle. Three hub targets were retrieved, including CENPF, CCNA2 (cyclin A), and CCNB1 (cyclin B1), which were highly expressed in esophageal cancer and associated with prognosis. Moreover, amentoflavone, a promising drug candidate found by pharmacophore-based virtual screening, showed antiproliferative and proapoptotic effects and induced G1 in esophageal squamous carcinoma cells. Taken together, our findings suggested that amentoflavone could be a potential cell cycle inhibitor targeting cyclin B1, and is therefore expected to serve as a great therapeutic agent for treating esophageal squamous cell carcinoma.

## Introduction

As the main subtype of esophageal carcinoma, esophageal squamous cell carcinoma (ESCC) is characterized by invasiveness, recurrence and ability to metastasize [1]. In addition, ESCC accounts for 90% of all cases of esophageal carcinoma in Asian countries. Chemoradiotherapy is one of the main treatment modalities for ESCC. However, significant side effects of chemoradiotherapy strategies persist as important factors creating dissatisfaction with the treatment. Although the U.S. Food and Drug Administration (FDA) recently approved the PD-1 immune checkpoint inhibitor pembrolizumab for use in combination with platinum-containing chemotherapy for treating unresectable esophageal cancer, there is still a lack of precise and effective therapeutic options, especially small molecule inhibitors [2].

With the development of gene microarray and high-throughput next-generation sequencing, the research of molecular mechanisms based on bioinformatics analysis has become an effective tool in cancer prevention [3]. With the help of bioinformatics, it is easier to discover novel biomarkers and drug targets for the diagnosis and treatment of diseases. The cell cycle is abnormal in most human tumors and causes the absence of differentiation and aberrant cell growth. Furthermore, deregulation of the cell cycle was shown to occur in the majority of patients with esophageal cancer [4]. Cell cycle was mainly tuned by a number of factors including cyclins and cyclin-dependent kinases (CDKs). In the cell cycle, cyclin B1, as a G2 phase cyclin, activates and forms a complex with CDK1, which promotes the G2/M phase transformation of cells. Also, cyclin A binds to CDK2 and is activated during G1/S transformation, thus regulating cell proliferation. Previous studies have shown that a significant difference for cyclin D1 expression was found between esophageal carcinomas and the adjacent epithelia and inhibiting cyclin D1 can enhance the radiosensitivity of esophageal cancer cells [5]. Based on the existing research, cyclin proteins such as cyclin A and cyclin B1 may also play an important role in the development of ESCC and remain to be explored in ESCC. Targeting cyclin may be an effective method to prevent the growth of ESCC.

In this study, we screened key targets related to ESCC through integrated bioinformatics analysis to seek out potential inhibitors. For this purpose, we first screened differentially expressed genes (DEGs) in ESCC. Then, Kyoto Encyclopedia of Genes and Genomes analysis revealed that the upregulated genes in ESCC were mainly associated with the cell cycle. Three targets were further identified, including CENPF, CCNA2 (cyclin A), and CCNB1 (cyclin B1). Moreover, based on pharmacophore-based virtual screening and molecular modeling, amentoflavone was found to exert a potential cyclin B1 targeting capability. Finally, experiments in vitro were adopted to further confirm the anticancer activity of amentoflavone in ESCC cell.

## Materials and Methods

### Identification of Differentially Expressed Genes

Differentially expressed genes (DEGs) were obtained from TCGA and GEO databases, respectively [6, 7]. The differential expression of mRNAs from TCGA was analyzed by Limma package (version: 3.40.2) of R software. The GSE70409 (GSM1727130 -GSM1727163) and GSE45670 (GSM1111665-GSM1111699) datasets were downloaded from the GEO database, and the GEO_2_R online analysis tool was applied to evaluate the DEGs between ESCC and normal tissue samples. Full details of patients can be found in the original publication of datasets [8, 9]. The points with |log FC| ≥ 1 and *p* < 0.05 were defined as statistically significant. Venn diagram analysis was used to overlap the DEGs between the TCGA and GEO datasets.

### Gene Ontology Analysis and Kyoto Encyclopedia of Genes and Genomes Analysis

The KOBAS8 database was used to perform GO enrichment and KEGG pathway analysis. The GO and KEGG enrichment results were extracted from KOBAS, and plotted using the statistical programming language R.

### Construction of Protein-Protein Interaction Network

Protein-protein interactions of the DEGs were constructed using the STRING database [10]. Following that, chart information was downloaded and beautified by Cytoscape [11]. In the generated network, every target has been indicated using nodes, and the interactions have been indicated using edges.

### Hub Target Analysis

CytoHubba [12], a plugin of Cytoscape, was used to identify targets possessing higher degrees using different computation methods including closeness, MCC, Degree, EPC, and NNC. The common targets that ranked in the top 10 of different screen results were considered hub targets. The expression of hub targets in esophageal cancer and esophageal squamous cell carcinoma was analyzed via GEPIA [13] and TCGA databases, respectively. Tumoral RNA-seq data were obtained from the Genomic Data Commons (GDC) data portal (82 of the tumors had mRNA expression data of paired normal tissue samples). All details, including donor gender, age range, biospecimen procurement methods, and sample fixation, were listed in GTEx official annotation. *p*-value of < 0.05 was considered statistically significant.

### Pharmacophore-Based Virtual Screening

The pharmacophore-based screening was performed by the online sever Pharmit [14]. The pharmacophore models were constructed according to the existing reports on CDK1/cyclin B1 complex (6GU2) and CDK2/cyclin A complex (4BCQ), which co-crystallized with flavopiridol and a 2-amino-4-heteroaryl- pyrimidine inhibitor, respectively. Compounds with a score <-7 were selected.

### Molecular Docking and Molecular Dynamics Simulation

Molecular docking was performed using AutoDock Vina software [15]. The crystal structures of 6GU2 and 4BCQ were retrieved from the Protein Data Bank [16]. All ligands and receptors were prepared using AutoDock Tools [17]. The docking box was defined as the center based on the original ligands, with a radius of approximately 30-50 Å. The poses of the compounds with the best binding affinity to the targets were generated using PyMOL. The AmberTools14 program was used for molecular dynamics (MD) simulations of the selected docked poses [18]. Moreover, AMBER FF14SB force field and the general AMBER force field (GAFF) were applied to calculate the force field parameters for proteins and ligands. The tleap module of AmberTools14 was used for system setup. The optimized structures of ligands were calculated using the Gaussian09 program with B3LYP/6-31G basis sets. The whole system was solvated in TIP3P water molecules in a box with dimensions of 10Å×10Å×10Å. Before the MD simulation, 2,500 steps of energy minimization were performed using the steepest descent and conjugate gradient method, respectively. Subsequently, constraints were released and the same 5,000 steps of energy minimization were then run for the entire system. During the MD simulations, the particle mesh Ewald (PME) method was performed to deal with the long-range electrostatic interactions. A non-bonded interaction cutoff of 10Å was employed. Using constraints at a constant volume, the entire system was heated from 0 K to 300 K within 60 ps, and then the solvent density was balanced under a stable system (T = 300 K, P = 1 atm) and sampled for 100 ns.

### Reagents and Cell Culture

Amentoflavone was purchased from Bide Pharmatech Ltd. (China). The human esophageal squamous carcinoma cell lines KYSE-150 and Eca-109 were purchased from the Institute of Biochemistry and Cell Biology, Chinese Academy of Sciences (China). The cells were cultured using RPMI-1640 (Gibco, Germany) medium, which was supplemented with 10% fetal bovine serum (FBS) and 1× antibiotic/antimycotic (Gibco).

### Cell Viability Assay

The cell viability was assessed by the MTT assay. Cells (4,000 cells/well in 96-well plates) were treated with amentoflavone and incubated at 37°C for 48 h. Then, thiazolyl blue (MTT) solution was added and incubated with cells for another 4 h. Cell viability was detected using a spectrophotometer (DTX880, Beckman Coulter, USA) at 490 nm.

### Colony Formation Assay

Cells (1000 cells/well) were seeded in 12-well adhesive culture plates. After cell attachment, cells were treated with different concentrations of amentoflavone and cultured for 15 days at 37°C. Then, colonies were washed with PBS and fixed with 4% paraformaldehyde and stained with 0.04% crystal violet. The number of colonies was then counted.

### EdU Staining Assay

For EdU staining, cells were seeded in 6-well plates and the EdU Kit (Beyotime, China) was used. Cells were incubated with amentoflavone for 48 h, and an EdU working solution was added to the plate. After 2 h, cells were treated with Click-iT Edu reagents. The EdU-positive cells were observed under a microscope.

### Cell Apoptosis Analysis

The FITC Annexin V Apoptosis Detection Kit (BD Biosciences, USA) was used for apoptosis analysis as previously described [34]. The cells treated with amentoflavone were harvested and resuspended in 1× binding buffer. Then, the cell suspension was incubated with propidium iodide and FITC Annexin V. After 15 min, staining was terminated with 400 μl of 1× binding buffer. Finally, the cells were analyzed using flow cytometry (BD Biosciences).

### Cell Cycle Distribution Analysis

Cells were cultured in 6-well plates and treated with amentoflavone for 48 h. After that, cells were harvested and fixed with 70% ethanol at 4°C for 24 h. Next, the cells were washed with PBS and incubated with RNase solution (100 μg/ml in PBS) for 30 min at room temperature. The cell cycle distribution was assessed using PI staining and detected by a FACSalibur flow cytometer (BD Biosciences).

### Transwell Assay

Transwell Permeable Supports, Polycarbonate Membranes (Corning Inc., USA) were used for transwell assay as previously described [19]. After treatment with amentoflavone (0, 50, 100, 150 μM) for 48 h, the cells were resuspended in serum-free medium (1 × 10^6^ cells/ml). Then, the cells were plated in Matrigel-coated (50 μl) Transwell inserts. Medium with 10% FBS was added into the lower chamber, and into the upper chamber, 100 μl of the cell suspension was added. After 48 h incubation, each transwell membrane was fixed with 4%paraformaldehyde and stained with 1% crystal violet. Following that, 33% (v/v) glacial acetic acid was used to dissolve crystal violet and absorbance was detected under 490 nm.

### Cell Adhesion Assay

For the cell adhesion assay, human fibronectin (2.5 μg/ml in PBS) was used to coat a 96-well plate. After treatment with amentoflavone for 48 h, cells were harvested and seeded into the 96-well plate in serum-free medium (4 × 10^4^ cells/well). The medium was removed after 1 h incubation at 37°C. Crystal violet was then used to stain the attached cells and the absorbance was measured at 560 nm.

### Western Blot

After being treated with amentoflavone for 48 h, the cells were lysed and boiled for 10 min. Subsequently, proteins were separated by SDS-PAGE and then transferred to a polyvinylidene fluoride (PVDF) membrane. After being blocked in 5% dry milk in Tris-buffer saline Tween (TBST) for 1.5 h and washed with 1× TBST, the PVDF membrane was treated with the following primary antibodies purchased from Beyotime (China) or Cell Signaling Technologies (USA): Bax, Bcl-2, cyclin B, cyclin A, CDK2 and CDK1. The ECL western blotting detection reagents were purchased from Thermo Fisher Scientific, and further analysis was conducted in ChemiDoc MP (BioRad).

### Statistical Analysis

Data were analyzed using a 2-tailed, unpaired Student’s *t*-test. The *p*-values are indicated in the figures (*p < 0.05; **p < 0.01; ***p < 0.001).

## Results

### Identification of DEGs

**Fig. 1:**
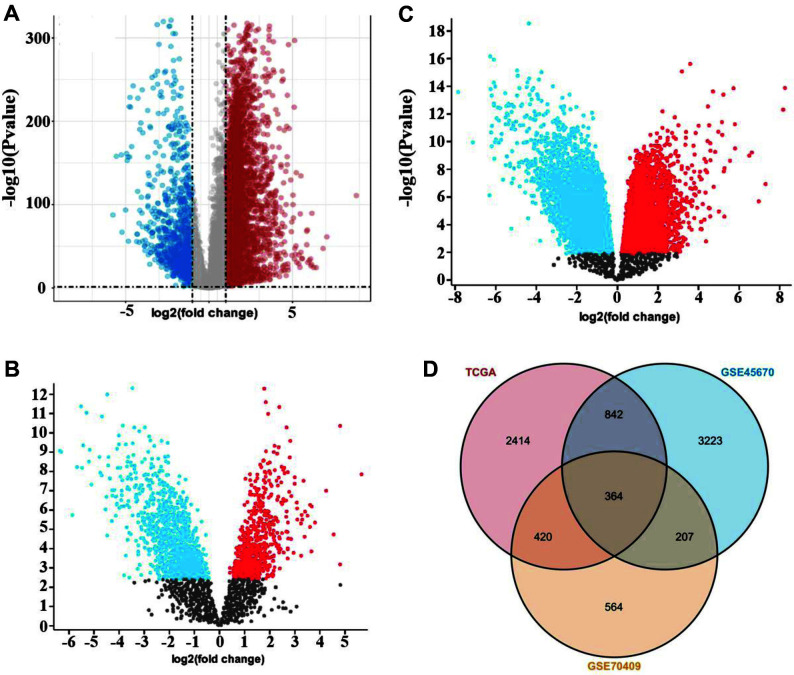
The differentially expressed genes (DEGs) in ESCC. (**A**) The volcano plot showed the number of DEGs based on the TCGA database. (**B** and **C**) The volcano plot showed the number of DEGs based on GSE70409 and GSE45670 datasets, respectively. (**D**) Venn diagram depicted overlaps among differentially expressed genes in TCGA and GEO.

DEGs in ESCC were retrieved based on the TCGA and GEO databases, respectively. According to the results from TCGA, 4,049 DEGs were identified, including upregulated genes and downregulated genes (Fig. 1A). Additionally, the GEO_2_R tool was utilized to analyze the DEGs in GSE70409 and GSE45670. Comparing the mRNA expression between ESCC and normal tissues, 1,555 and 4,636 DEGs were screened out from the two datasets, respectively (Figs. 1B and 1C). Moreover, by overlapping the DEGs from TCGA and GEO using Venn diagram analysis, a total of 364 common DEGs were found which were considered as significant changes (Fig. 1D).

### Functional and Pathway Enrichment Analysis of the DEGs

**Fig. 2:**
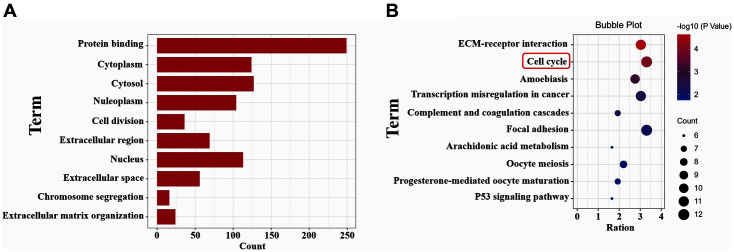
Go and KEGG analysis of DEGs. (**A**) The top 10 enriched GO terms. (**B**) The top 10 enriched KEGG terms.

Further, GO and KEGG analyses were performed using 364 common DEGs, and the top 10 significantly enriched terms were shown in Fig. 2. The gene ontology term results included protein binding, cytoplasm, cytosol, nucleoplasm, cell division (Fig. 2A). The high-ranked KEGG terms included ECM-receptor interaction, cell cycle, amoebiasis, transcriptional misregulation in cancer, and complement and coagulation cascades (Fig. 2B). Notably, the cell cycle was one of the most significant results with the smallest *p*-value. The results suggested that common DEGs were mainly involved in the cell cycle.

### Construction of Protein-Protein Interaction Network

**Fig. 3:**
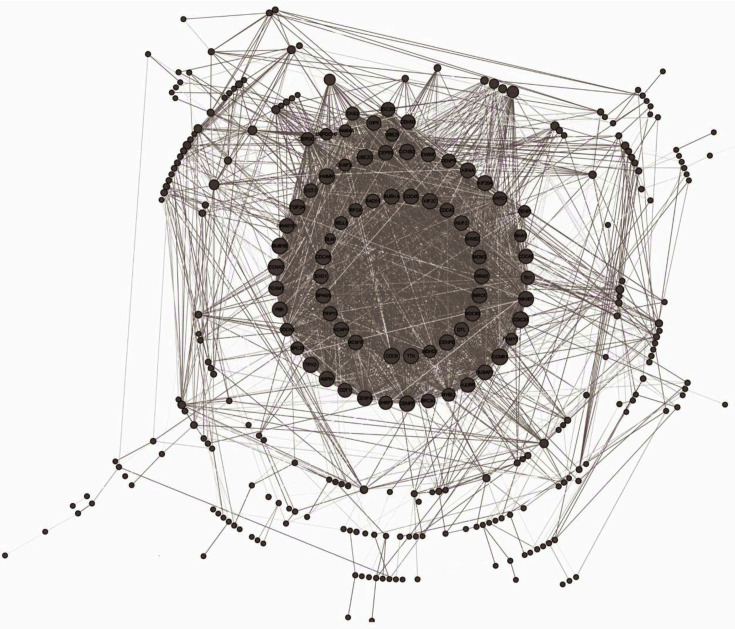
The PPI network of DEGs.

Following the prediction by STRING, we constructed a protein-protein interaction (PPI) network for the overlapping DEGs using Cytoscape, which included 291 nodes and 3119 edges (Fig. 3). Each node represents a target, and node size is proportional to the degree of connectivity with other targets. We observed that some targets involving in the cell cycle such as CDC6, CDCA8, CDC20, and CCNB1 had higher degree values.

### Hub Target Analysis

**Fig. 4:**
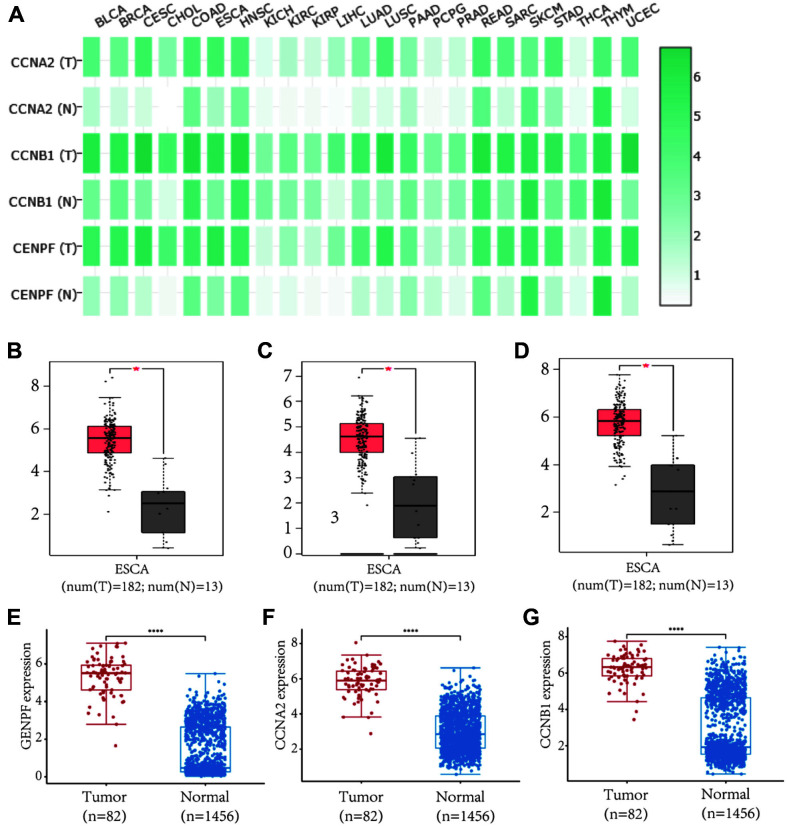
Verifying the mRNA expression of hub genes including CCNA2, CCNB1, CENPF. (**A**) The mRNA expression of hub genes in different cancer types and normal samples. T represents tumor tissues and N represents normal tissues. (**B-D**) The mRNA expression of CENPF (**B**), CCNA2 (**C**), CCNB1 (**D**) in esophageal carcinoma. (**E-G**) The mRNA expression of CENPF (**E**), CCNA2 (**F**), CCNB1 (**G**) in esophageal squamous cell carcinoma. *p < 0.05 was considered statistically significant.

After constructing the PPI network, hub targets were selected from the PPI network using cytoHubba. As shown in Table 1, different algorithms including Closeness, MCC, Degree, EPC, and NNC were used to calculate the top 10 hub targets. CCNB1 (cyclin B1), CCNA2 (cyclin A), and CENPF were three common targets that ranked in the top 10 of all calculated results. The GEPIA database was used to analyze the expression of the three hub targets in esophagus cancer and normal tissues. As shown in Fig. 4A, green color represents higher expression while white represents lower expression. Cyclin B1, cyclin A, and CENPF were overexpressed in several tumor types including esophageal carcinoma, bladder urothelial carcinoma, breast cancer, and colon adenocarcinoma. The mRNA level of these three hub targets was significantly higher in esophagus cancer tissues than it was in adjacent normal tissues (Figs. 4B and 4D). Further analysis based on TCGA revealed that these three hub targets were also overexpressed in ESCC (Figs. 4E and 4G).

### Prediction of Targeted Drugs

Currently, no cocrystal structures of CENPF with any small molecule are available. Only the cocrystal structures of CDK1/cyclin B1 and CDK2/cyclin A complexes have been reported. Thus, we further performed pharmacophore-based virtual screening based on the related cocrystal structure of cyclin B1 and cyclin A. According to the CDK1/cyclin B1 complex binding with flavopiridol (6GU2), the generated model consisted of two hydrophobic features, three hydrogen bond acceptors, and three hydrogen bond donors (Fig. S1A). For CDK2/cyclin A complex binding with a 2-amino-4-heteroaryl-pyrimidine inhibitor (4BCQ), the generated model consisted of one hydrophobic feature, two hydrogen bond acceptors, and two hydrogen bond donors (Fig. S1B). Virtual screening of cyclin B1 and cyclin A based on MolPort library resulted in 123 and 164 hits, respectively, with the top 10 results being listed in Table 2. The compounds that hit CDK1/cyclin B1 and CDK2/cyclin A with best score were amentoflavone (MolPort-001-741-078) and 3-Hydroxy-2-(3,5,7-trihydroxy-4-oxo-4H-chromen-2-yl)phenyl β-D-glucopyranoside (MolPort-019-937-505), respectively. Their chemical structure and other representative compounds were shown in Fig. S2.

**Fig. 5:**
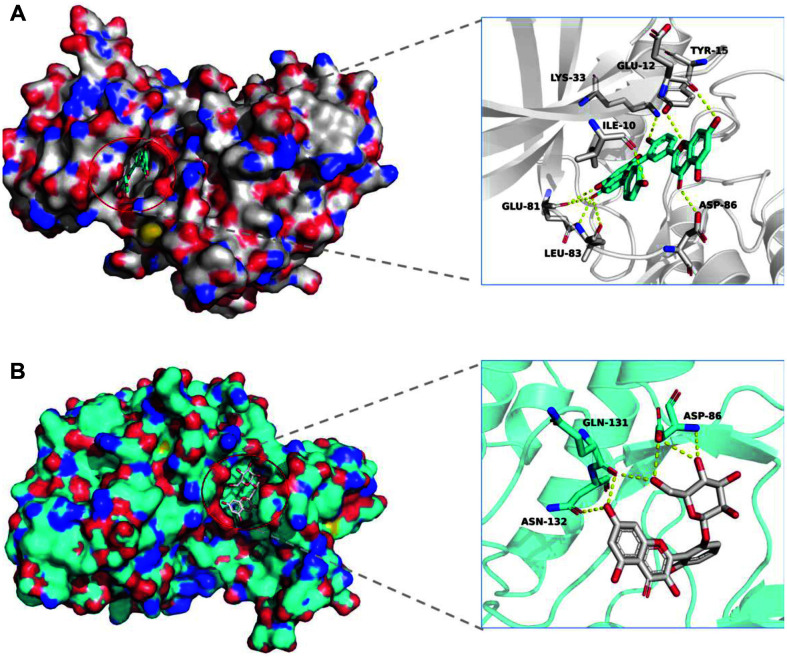
Molecular Docking. (**A**) Calculated binding mode of amentoflavone with CDK1/cyclinB complex. (**B**) Calculated binding mode of 3-Hydroxy-2-(3,5,7-trihydroxy-4-oxo-4H-chromen-2-yl)phenyl β-D-glucopyranoside with CDK2/cyclin A complex.

**Fig. 6:**
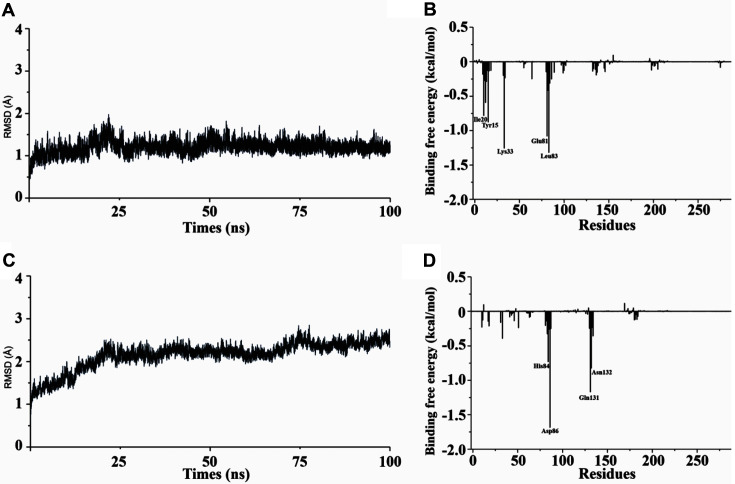
The analysis from 100 ns molecular dynamics simulations highlighting the conformational stability through root-mean-square deviation (RMSD; left panel) and total intermolecular contact (right panel) analyses for CDK1/cyclin B complex (A and B) and CDK2/cyclin A complex (C and D).

Moreover, molecular docking was performed to further investigate interactions between best-hitting compounds and targets via AutoDock Vina. Fig. 5A shows the optimal conformation of amentoflavone binding with CDK1/cyclin B1 complex, which resulted in a low binding energy of −10.3 kcal/mol. Moreover, Asp86, Leu83, Glu81, Lys33, Tyr15, Glu12, Ile10 were the key amino acid residues in hydrogen binding interaction between CDK1/cyclin B1 complex and amentoflavone. The other molecular docking result also showed that the No. 1 top hit compound can effectively bind with CDK2/cyclin A complex (Fig. 5B), which resulted in a low binding energy of -9.7 kcal/mol. The key hydrogen bonds with the amino acid residues of Asp86, Gln131, Asn132 were formed. Then, additional molecular dynamics simulations (100 ns) were adopted to further validate the results of the molecular docking. Initially, the root-mean-square deviation (RMSD) was calculated by comparing with the initial position of complexes which showed that the CDK1/cyclin B1-amentoflavone interaction displayed an RMSD of ~1.0 at 100 ns into the simulation (Fig. 6A) while the CDK2/cyclin A-glucopyranoside interaction displayed an RMSD of ~2.0 (Fig. 6C). Notably, CDK1/cyclin B1 complex has a small fluctuation before 20 ns, and then tends to stabilize. Overall, the complexes were stable and retained their structure during the simulation. To explore the binding affinity of each ligand, the MM/PBSA approach was used to perform binding free energy calculations on the complexes. The results showed that electrostatic interaction (ΔEele) was a major interacting force between ligands and receptors, comparing to the van der Waals interaction energy (ΔEvdW) (Table 3). Moreover, both the cyclin A and cyclin B1 complexes exhibited low ΔGTot values (-22.2 ± 0.2 and -25.18 kcal/mol, respectively). To further study the key amino acid residues in protein binding, we calculated the energy decomposition of amino acid residues based on MM/PBSA free energy. As shown in Fig. 6B, several residues including Ile10 (-0.78 kcal/mol), Tyr15 (-0.87 kcal/mol), Lys33 (-1.25 kcal/mol), Glu81 (-1.08 kcal/mol), and Leu83(-1.32 kcal/mol) were identified that exhibited significant energetic contributions to the stability of cyclin B1 complex. The energy decomposing result of cyclin A complex revealed that the major contribution came from His84 (-0.73 kcal/mol), Asp86 (-1.68 kcal/mol), Gln131 (-1.16 kcal/mol) and Asn132 (-0.82 kcal/mol) (Fig. 6D). These results were consistent with the previous docking results, which suggested that amentoflavone may target cyclin B1.

### Validation of the Anti-Tumor Activities of Amentoflavone

**Fig. 7:**
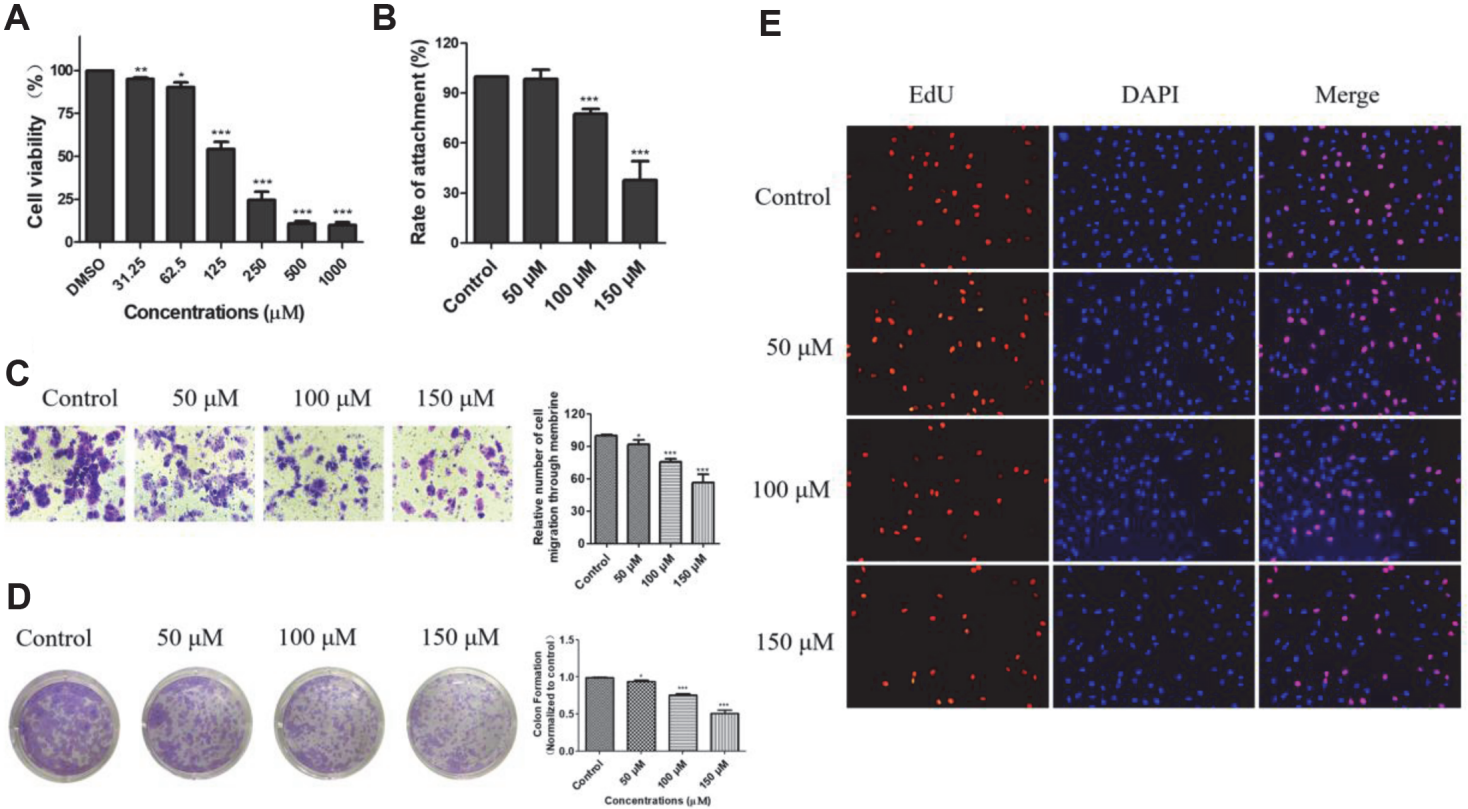
Anti-cancer capabilities of amentoflavone. (**A**) Cells were treated with different concentrations of amentoflavone for 48 h, then an MTT assay was conducted to detect cell viability. (**B**) The adhesion assay was conducted to test the adhesion ability of KYSE-150 cells. (**C**) The transwell assay was conducted to detect the invasive capacity of KYSE-150 cells. (**D**) The clonogenic assay was performed to detect the colony-forming ability of KYSE-150 cells. (**E**) Number of EdU-positive KYSE-150 cells after treatment with amentoflavone. In the bar chart, the data represent mean ± SD (*n* = 3). *p < 0.05, **p < 0.01 and ***p < 0.001 compared to the control group.

To identify the potential anti-cancer activity of amentoflavone, we explored the effects of amentoflavone on esophageal squamous cancer cells in vitro. Cells were treated with amentoflavone at various concentrations, and an MTT assay was performed to detect cell viability. The MTT result showed that amentoflavone effectively suppressed the cellular proliferation of the KYSE-150 and Eca-109 cells in a dose-dependent manner (Figs. 7A and 9A). Furthermore, we also tested the cytotoxicity of amentoflavone against normal HEEC cells. As shown in Fig. 9B, amentoflavone had a less cytotoxic effect on normal HEEC cells compared with cancer cells. Moreover, a reduced number of KYSE-150 clones was observed in the clone formation assay (Fig. 7D). To further test the antiproliferative effect of amentoflavone on KYSE-150 cells, an EdU assay was conducted. As shown in Fig. 7E, the number of EdU-positive cells decreased significantly after treatment with amentoflavone. In addition, further experiments were performed on KYSE-150 cells to explore the effect of amentoflavone on cell adhesion and invasion. As a result, amentoflavone was shown to significantly decrease the number of adherent cells in the adhesion assay (Fig. 7B). Also, as illustrated in Fig. 7D, amentoflavone significantly reduced the number of invasive cells; only 51.2% of invading cells were observed with the highest drug concentration used (Fig. 7C). Therefore, these findings indicated that amentoflavone has an inhibitory effect on cancer cell proliferation, invasion, and adhesion.

### Amentoflavone Induces Cell Apoptosis and Cell Cycle Arrest in Esophageal Squamous Cancer Cells

**Fig. 8:**
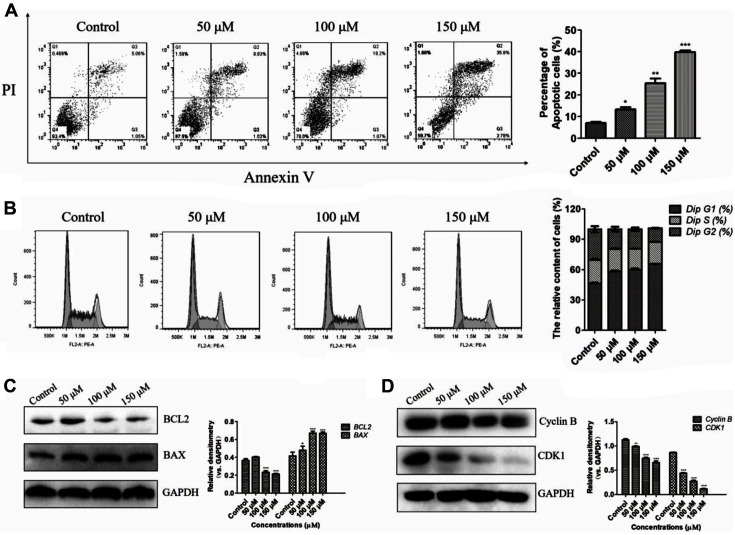
Amentoflavone induced apoptosis and cell cycle arrest in KYSE-150 cells. (**A**) Cells were treated with amentoflavone (50, 100, and 150 μM) for 48 h, then apoptosis analysis was determined by flow cytometry. (**B**) Cell cycle distribution analysis was examined by flow cytometry. (**C**) Representative western blot images and quantitative analysis of BAX and BCL-2 proteins. (**D**) Representative western blot images and quantitative analysis of cyclin B and CDK1 proteins. Values indicate the mean ± SD of three independent experiments. *p < 0.05, **p < 0.01 and ***p < 0.001 compared to the control group.

**Fig. 9:**
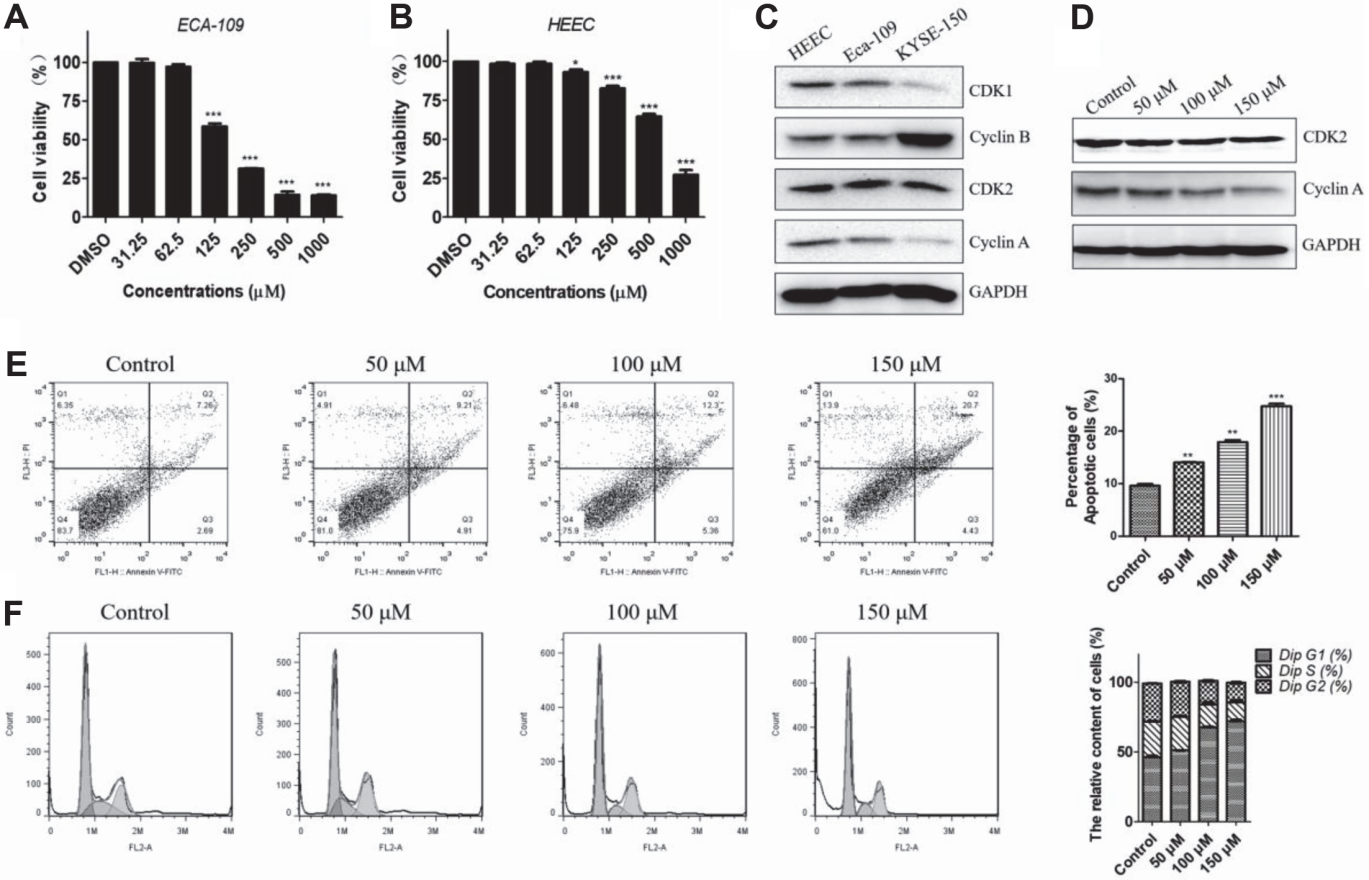
The effect of amentoflavone on Eca-109 and HECC cells. (**A** and **B**) Eca-109 and HEEC cells were treated with amentoflavone for 48 h, then MTT assay was conducted to detect cell viability. (**C**) The expression level of CDK1, cyclin B, CDK2, cyclin A in different cells. (**D**) KYSE-150 cells were treated with amentoflavone for 48 h, then western blotting analysis was used to detect the expression of CDK2 and cyclin A proteins. (**E**) Eca-109 cells were treated with amentoflavone (50, 100, and 150 μM) for 48 h, then apoptosis analysis was determined by flow cytometry. (**F**) Cell cycle distribution analysis of Eca-109 cells was examined by flow cytometry. Values indicate the mean ± SD of three independent experiments. *p <0.05, **p < 0.01 and ***p < 0.001 compared to the control group.

Flow cytometry and western blotting analyses were performed to further explore whether amentoflavone induced apoptosis and cell cycle arrest. Protein expression levels varied among the different cell lines, as shown in Fig. 9C, and high expression of cyclin B protein was observed in KYSE-150 cells. Moreover, according to the flow cytometry analysis, amentoflavone dose-dependently induced apoptosis of KYSE-150 and Eca-109 cells (Figs. 8A and 9E). Consistent with flow cytometry data, the western blotting also showed that amentoflavone treatment increased the expression of BAX and decreased the expression of Bcl-2 (Fig. 8C). Additionally, as amentoflavone is considered to have CDK1/cyclin B complex-targeting ability, the KYSE-150 cells treated with amentoflavone showed a decreased expression of cyclin B and CDK1 (Fig. 8D). Also, as shown in Figs. 8B and 9F, the flow cytometry analysis indicated that amentoflavone resulted in a significant increase in the percentage of cells in the G1 phase. The expression of cyclin A and CDK2 was also suppressed in KYSE-150 cells (Fig. 9D). Altogether, these results demonstrate that amentoflavone effectively induces apoptosis and G1 phase arrest in KYSE-150 cells.

## Discussion

ESCC is the predominant pathological type of esophageal cancer. Due to a lack of early diagnosis and the limitations of conventional treatment strategies, clinical outcomes of ESCC are not yet satisfactory [20]. At present, the main treatment strategies for ESCC are surgery supplemented by radiotherapy and chemotherapy [21]. Although advances have recently been made in identifying the genomic drivers of esophageal cancer, there is still a lack of effective targeted therapies. Moreover, both in esophageal squamous cell carcinoma and esophageal adenocarcinoma, advanced-stage patients are commonly observed [22]. Thus, discovering new molecular indicators and novel targeted therapeutics is of great clinical significance for the early diagnosis and treatment of ESCC. Under these circumstances, this work explores the molecular characterization and potential therapeutic candidates of ESCC, with the goal of assisting in the development of personalized targeted therapies.

In this study, we collected differential expression data from TCGA and GEO databases. Then, 364 common DEGs were screened out by Venn analysis. Meanwhile, the KEGG analysis of 364 DEGs suggested that the cell cycle was one of the main enriched pathways. Our results indicated that cell cycle-related proteins may play key roles in the process of ESCC. The abnormal cell proliferation under cell cycle disorder contributes a lot to tumorigenesis [23]. Previous studies have suggested that cyclin D1 is amplified in 54% and overexpressed in 63%of squamous cell-type tumors [24]. Interference in cell cycle control shows promise for inhibiting the progress of ESCC, and indeed, some studies have revealed that CDK4/CDK6 inhibitors achieved good results against ESCC [25, 26]. Furthermore, we constructed a PPI network based on 364 common DEGs and screened the hub targets via different methods. Three targets, including CENPF, CCNB1 (cyclin B1), CCNA2 (cyclin A), were hit at a high frequency. Cyclin B1 and cyclin A are known as key regulators of the cell cycle. As the regulatory subunit of the kinase CDK1, cyclin B1 protein forms an active complex with CDK1 and is mainly involved in the regulation of the G2/M phase transition. However, previous research indicated that decreased expression of cyclin B1 was also observed when G1 phase arrest occurs [27, 28], which was similar to our results. Cyclin A is a cyclin protein that participates in multiple links of the cell cycle, associating with CDK2 and CDK1 and further activating both of them [29]. The cyclin A/CDK2 complex is necessary for passage into the S phase, while the association with CDK1 is indispensable for entry into the M phase [30]. Previous study has suggested that cyclin A immunopositivity correlates with cancer risk and that cyclin A could be used as a biomarker to identify Barrett's esophagus patients who have a higher risk of progression [31]. The other hub target CENPF is involved in chromosome segregation during cell division and is part of the nuclear matrix during the G2 phase of the cell cycle [32, 33]. These findings supported the pursuit of these hub targets related to the cell cycle as biomarkers or potential therapeutic targets of ESCC. Given the tight linkage between cyclin B1 and CDK1, and cyclin A and CDK2, we then chose the reported cocrystal structure of CDK1/cyclin B1and CDK2/cyclin A to seek potential targeting drugs. Compared with the docking-based virtual screening (DBVS), it is more reliable to find novel targeted drugs based on pharmacophore-based virtual screening (PBVS) [34]. So, the PBVS method was used to screen effective drugs that targeted the CDK1/cyclin B1and CDK2/cyclin A complexes; further, molecular docking and molecular dynamics simulations were used to validate their potential binding. Fortunately, we found some interesting compounds that may exert inhibitory activity on cell cycle-related proteins such as amentoflavone. Most of the hit compounds we found have not been reported as cell cycle inhibitors in ESCC. Based on the results of virtual screening, amentoflavone was selected to perform biological experiments to further confirm its anti-tumor activity in esophageal squamous cancer cells. Our results showed that amentoflavone effectively inhibited proliferation, invasion, and adhesion of esophageal squamous cancer cells in vitro. Moreover, as expected, amentoflavone can significantly suppress the expression of cyclin B and CDK1, indicating that this biflavonoid may directly or indirectly target CDK1/cyclin B1 complex.

In summary, our results demonstrated an effective approach to integrate bioinformatics, structure-based virtual screening and biological evaluation, and clearly point to a potential molecular-targeted drug for treating ESCC. We found that cyclin A and Cyclin B1 were involved in the progress of ESCC with the help of integrated bioinformatics analysis. Cell-cycle regulatory proteins cyclin B1 and cyclin A were found to be highly expressed in ESCC. Structure-based virtual screening indicated that amentoflavone may exert its potential anti-cancer effects via targeting cyclin B1/CDK1 complex which was further validated by in vitro experiments. Finally, the present study provides new insight into ESCC and proposes amentoflavone-related therapies for this disease.

## Supplemental Materials

Supplementary data for this paper are available on-line only at http://jmb.or.kr.

## Figures and Tables

**Table 1:** The top 10 hub genes were screened out using different method by cytoHubba.

	Method	Closeness	MCC	Degree	EPC	NNC
Rank						
1		CCNB1	CCNB1	CCNB1	CCNB1	CCNA2
2		CCNA2	CENPF	CCNA2	CENPF	CDC20
3		MKI67	ASPM	CDC20	ASPM	CCNB1
4		FOXM1	CDC6	BUB1B	TRIP13	BUB1B
5		CDC20	NUF2	UBE2C	CDC6	UBE2C
6		UBE2C	CCNA2	CDC6	AURKA	TOP2A
7		PBK	BUB1B	TOP2A	MYBL2	CDC6
8		KIAA0101	CCNB2	CENPE	OIP5	CENPE
9		CEP55	TPX2	CCNB2	NCAPH	CCNB2
10		CENPF	CDC45	KIF2C	GINS2	KIF2C

**Table 2:** Pharmacophores-based virtual screening identified hit compounds against CDK1/Cyclin B1 and CDK2/ Cyclin A.

Rank	CDK1/Cyclin B1 (6GU2)	Common name	Score	CDK2/Cyclin A (4BCQ)	Score
1	MolPort-001-741-078	Amentoflavone	-11.95	MolPort-019-937-505	-8.74
2	MolPort-005-945-277	Agathisflavone	-11.27	MolPort-047-395-878	-8.49
3	MolPort-001-742-110	2,3-Dihydroamentoflavone	-11.06	MolPort-028-604-163	-8.40
4	MolPort-035-705-955	Putraflavone	-10.59	MolPort-004-835-422	-8.39
5	MolPort-046-785-897	Orientin	-10.23	MolPort-007-819-413	-8.35
6	MolPort-001-742-110	Xanthomicrol	-10.20	MolPort-046-853-755	-8.34
7	MolPort-047-483-636	8-C-Galactosylluteolin	-10.11	MolPort-046-836-416	-8.25
8	MolPort-009-752-656	Gossypin	-10.07	MolPort-000-469-270	-8.17
9	MolPort-003-935-138	Vitexin	-10.05	MolPort-002-578-947	-8.13
10	MolPort-046-785-897	Ganoderic acid D	-9.97	MolPort-004-835-422	-8.12

The top 10 hit compounds and their screening score were listed.

**Table 3:** The results of MM/PBSA free energy calculation (kcal/mol).

Energy Component	ΔEvdw	ΔEele	ΔGTot
Cyclin A complex	-28.56	-31.06	-23.89
Cyclin B complex	-31.99	-34.85	-25.18
