# Supplementary material for: Discovery of Anticancer Activity of Amentoflavone on Esophageal Squamous Cell Carcinoma: Bioinformatics, Structure-Based Virtual Screening, and Biological Evaluation
Source: J Microbiol Biotechnol. 2022 May 4;32(6):718–29. doi: 10.4014/jmb.2203.03050 (PMC9628896; doi:10.4014/jmb.2203.03050)
Supplement: Supplementary file 1 [file jmb-32-6-718-supple.pdf]

J. Microbiol. Biotechnol.
https://doi.org/10.4014/jmb.2203.03050

Supplementary Figures

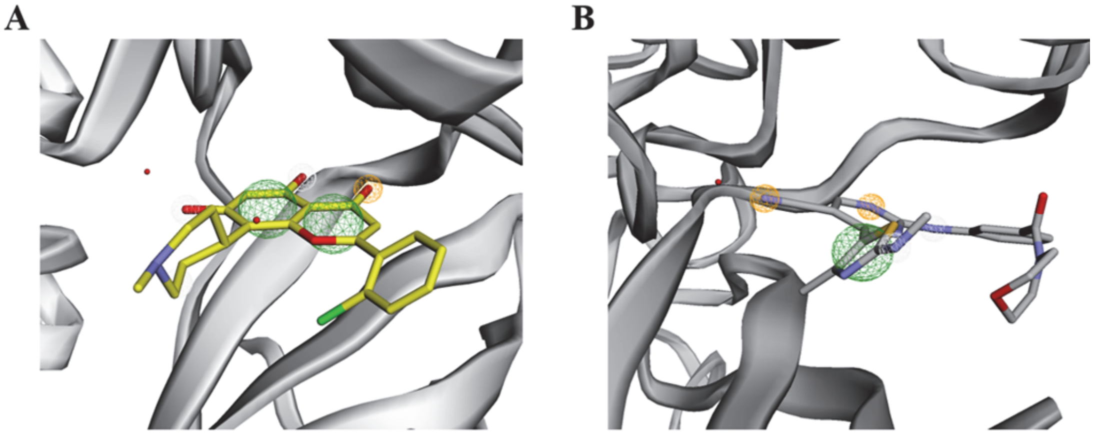

Fig. S1. pharmacophore models of virtual screening. **A** CDK1/Cyclin B1 complex binding with flavopiridol. **B** CDK2/Cyclin A complex binding with a 2-amino-4-heteroaryl- pyrimidine inhibitor.

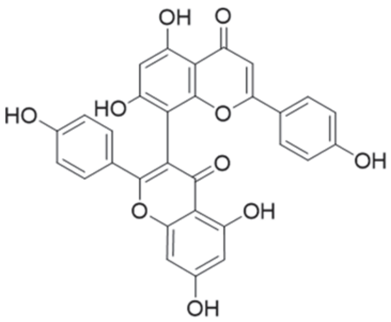

MolPort-001-741-078

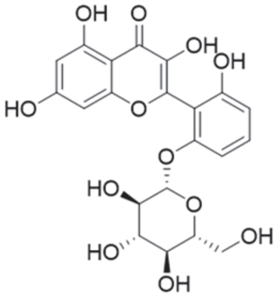

MolPort-019-937-505

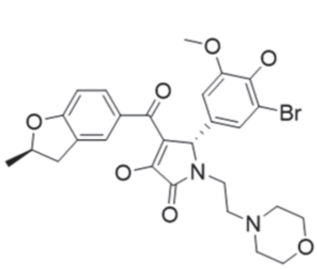

MolPort-004-835-422

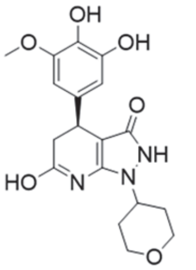

MolPort-028-604-163

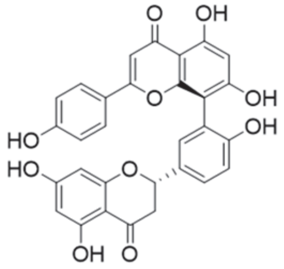

MolPort-001-742-110

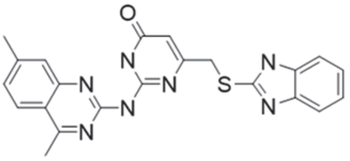

MolPort-000-469-270

Fig. S2. the chemical structure of representative compounds.
